# Immune-Competent Tumor Organoid Models: Construction Strategies and Their Application in Predicting Immune Checkpoint Blockade Response

**DOI:** 10.3390/cancers18162639

**Published:** 2026-08-15

**Authors:** Qi Zhang, Hong Zeng, Xueying Wan, Lei Lang

**Affiliations:** 1 Department of Clinical Laboratory, Jiangjin Hospital, Chongqing University of Chinese Medicine, Chongqing Jiangjin District Hospital of Chinese Medicine, Chongqing 402247, China; 2 Key Laboratory of Clinical Laboratory Medical Diagnostics (Chinese Ministry of Education), Chongqing Medical University, Chongqing 400016, China; 3 Department of Urology, Jiangjin Hospital, Chongqing University of Chinese Medicine, Chongqing Jiangjin District Hospital of Chinese Medicine, Chongqing 402247, China; 4 Department of Clinical Laboratory, Chongqing Emergency Medical Center, Chongqing University Central Hospital, School of Medicine, Chongqing University, Chongqing 400014, China

**Keywords:** tumor organoid, immune checkpoint blockade, air–liquid interface, tumor–immune co-culture, response prediction, tumor microenvironment

## Abstract

The immune checkpoint blockade (ICB) has changed the way many solid tumors are treated; yet, only a minority of patients respond durably. The biomarkers that guide therapy (the PD-L1 expression, tumor mutational burden, and microsatellite status) read only fixed molecular features and miss the shifting tumor–immune exchange. Patient-derived tumor organoids reproduce the tumor epithelium and predict the chemotherapy responses, but, in their usual form, they omit the immune compartment on which the ICB acts. This review asks how immune-competent organoids can close that gap, and how well they predict the response. One idea organizes the field: how a model is built determines what it can predict. Two complementary construction routes have been developed. One preserves endogenous immunity through an air–liquid interface culture; the other rebuilds it by a co-culture with defined effector populations. Both now incorporate microenvironmental reconstruction and quantitative functional readouts. The most developed examples are in colorectal cancer, with breast cancer applications only beginning to appear. Evidence for the ICB response prediction, graded on a four-level scale, remains at the proof-of-concept stage. Organoid functional assays complement rather than replace molecular biomarkers. Their clinical translation requires progress on four fronts: standardized protocols, longer immune cell viability, spatial and multi-omic integration, and prospective paired validation.

## Simple Summary

The immune checkpoint blockade has transformed the treatment of many solid cancers; yet, most patients gain no benefit, and current tests cannot reliably predict who will respond. Part of the reason is that these tests read only fixed molecular features and miss the living interaction between cancer cells and the immune system. Patient-derived tumor organoids are miniature tumors grown in the laboratory from a patient’s cancer, but, in their usual form, they contain no immune cells. This review describes how researchers are developing organoids that preserve or rebuild the immune compartment, allowing a patient’s tumor and immune cells to be observed as they fight each other in a dish. We explain how each model’s design shapes what it can predict, gather early evidence that such models can forecast the immunotherapy response, and set out the steps needed before they can guide treatment. The evidence so far is encouraging but preliminary.

## 1. Introduction

The immune checkpoint blockade (ICB) targeting the PD-1/PD-L1 axis now achieves durable responses in solid tumors that conventional cytotoxic therapy could not deliver. The effect is clearest in tumors with a mismatch repair deficiency (dMMR), where the PD-1 blockade achieves high and durable response rates. That result secured the first tissue-agnostic approval of a checkpoint inhibitor [1,2]. Across most solid tumors, however, the benefit reaches only a minority. In triple-negative breast cancer (TNBC), adding pembrolizumab to chemotherapy improved the pathological complete response and event-free survival in KEYNOTE-522; yet, a substantial fraction of patients gain no benefit while being exposed to immune-related toxicity [3,4]. Identifying which patients will respond before treatment remains an unmet clinical need.

The current companion biomarkers meet this need only in part. The PD-L1 immunohistochemistry is confounded by an antibody clone and cut-off heterogeneity and by variability within a tumor, with the predictive performance inconsistent between cancer types [5,6]. The tumor mutational burden and microsatellite instability enrich for responders but leave many unidentified, with thresholds differing by histology [2,7], and inflamed gene expression signatures correlate only weakly with mutational metrics and are not used routinely [8,9]. One limitation runs through all of these tools: each is a static molecular snapshot that cannot capture the functional dialog between a patient’s tumor and their immune system. These limitations are also unevenly distributed, concentrating in the microsatellite-stable, PD-L1-low, intermediate-mutational-burden tumors that make up most checkpoint non-responders and for which no reliable predictor exists. That subgroup, rather than the biomarker-enriched populations already served by companion diagnostics, is where a functional test would carry the greatest clinical value, and is the population against which the models reviewed here should ultimately be judged (Section 8).

Functional prediction needs a model that preserves that dialog, and conventional preclinical systems fall short: cell lines and patient-derived xenografts lack an autologous immune compartment, so the interactions governing the ICB response are absent [10]. Patient-derived tumor organoids (PDTOs) reproduce the genomic and histological features of their parental tumors, and they predict responses to chemotherapy and targeted agents [11,12,13,14]. In their conventional form, however, they are epithelial monocultures that have been stripped of the immune and stromal cells on which the ICB acts. This “immune gap” must be closed before organoids can serve as functional predictors of immunotherapy.

Two routes close it. An air–liquid interface (ALI) culture propagates tumor fragments without enzymatic dissociation, preserving endogenous tumor-infiltrating lymphocytes, myeloid cells, and native T-cell receptor repertoires alongside the epithelium [15]. Reconstitution instead adds exogenous effectors to expanded epithelial organoids, where the co-culture of PDTOs with autologous peripheral blood lymphocytes generates tumor-reactive T cells and a scalable functional readout [16,17]. Both have modeled the PD-1/PD-L1 blockade ex vivo, with early studies reporting organoid responses that track the checkpoint sensitivity and even reproduce the adaptive resistance through local regulatory T-cell expansion [18,19]. These findings remain at the proof-of-concept stage and are from small, mostly retrospective cohorts still lacking prospective paired validation against patient outcomes.

This review links construction to prediction. Existing reviews have largely treated organoid immune modeling and organoid-based response prediction as separate topics [20,21,22,23]. Here, a taxonomy of construction strategies is set beside their emerging application to ICB response prediction, so that how a model is built is tied explicitly to what it can predict. Colorectal and breast cancer serve as the principal examples, for different reasons. Colorectal cancer supplies most of the available ICB prediction data. Breast cancer combines a pressing clinical need with substantial engineering groundwork; yet, no peer-reviewed study has shown that a breast organoid can predict checkpoint response. Evidence across other solid tumors is surveyed alongside them, graded throughout on the four-level scale defined in Section 2. Rather than overstating organoids as validated predictors, the review sets out the standardization and prospective validation steps needed to move them from bench-side avatars toward clinical decision tools [24].

Reading these two bodies of work together yields three consequences neither gives alone. First, one explicit criterion is applied to the boundary between preservation and reconstitution: whether the immune compartment is separated during derivation and then reintroduced (Section 3). That criterion places the co-culture with autologous tumor-infiltrating lymphocytes on the reconstitution side. The autologous provenance therefore becomes an axis independent of the construction route. The effector-to-target ratio also emerges as a quantity that the investigator sets rather than a property of the tumor (Section 5). Second, culture conditions must be counted as part of construction. Two laboratories that follow the same nominal route with different media are therefore running different constructions. A medium composition, cytokine support, and the readout time point become reporting requirements. The parameters most often left unreported (Section 5) are the ones that decide whether two results can be compared. Third, the construction is mapped onto the predictive capability rather than tumor type, which makes the mapping format-specific. The one checkpoint-induced suppressive mechanism reported to date was observed in a preservation-based model. Reconstituted models instead address questions of effector potency and antigen specificity that preservation cannot (Section 4, Section 5 and Section 6).

## 2. Literature Search and Evidence Appraisal

Because the strength of the evidence is central to the argument, how it was gathered and appraised is set out here. PubMed/MEDLINE was the primary source, searched from inception to July 2026 and supplemented by the reference list screening of the retrieved reviews and Crossref look-ups. The searches were run as seven thematic blocks. The first set of terms covered the model (“organoid”, “patient-derived tumor organoid”, “PDTO”, “air–liquid interface”, “assembloid”, “tumor-on-chip”, and “microfluidic”). The second covered the immune compartment (“tumor-infiltrating lymphocyte”, “co-culture”, “immune microenvironment”, “cancer-associated fibroblast”, “regulatory T cell”, “CAR-T”, and “natural killer”). The third covered the clinical question (“immune checkpoint blockade”, “PD-1”, “PD-L1”, “response prediction”, and “immunotherapy efficacy”). No language filter was applied beyond English-language full-text availability.

Records were selected for the relevance to how an immune-competent tumor model is constructed, what functional readouts it supports, and whether those readouts have been related to the checkpoint inhibitor response. Priority went to work published between 2021 and 2026, with earlier studies retained where they established a method still in use or defined a concept the field relies on. Preclinical studies without an immune component, reports confined to non-malignant tissue, and studies of therapies other than the checkpoint blockade were cited only where needed for a specific methodological or contextual point, and are identified as such. Every cited record was checked against its PubMed or publisher entry, and each citation was checked to confirm that the source supports the statement it is attached to.

Evidence for the checkpoint response prediction is reported on a four-level scale used consistently in the text and in the evidence table of Section 7. “Feasibility” denotes a demonstration that a model can be built and exposed to a checkpoint agent, without a response-related endpoint. “Proof-of-concept” denotes a measured, checkpoint-dependent functional response in patient-derived material, with mechanistic and case-level variants noted where relevant. “Emerging” denotes a reported relationship between an ex vivo readout and a clinical response measure in a small or retrospective series. “Blank” denotes a setting where the clinical need is established but no peer-reviewed predictive demonstration exists. The scale grades how directly a study bears on the predicting response, not study quality, and is applied by narrative appraisal.

This appraisal is not a systematic review. It does not follow PRISMA and lacks the features that would define one: a registered protocol, dual independent screening and extraction, an exhaustive multi-database search with the reported yield at each stage, and a formal risk-of-bias instrument. It is, instead, a comprehensive narrative overview with an explicit, uniformly applied evidence scale. A selection bias toward positive and methodologically novel reports is therefore possible, which matters particularly where negative co-culture results are rarely published; where such a result was available, it has been included and identified.

## 3. The Immune Gap in Preclinical Tumor Models

Patient-derived tumor organoids keep the genetic and phenotypic identity of their parent tumors, which is why they have become a leading avatar for precision oncology. Yet, the same trait hides a structural blind spot: a standard organoid is an epithelial culture that leaves out the immune environment on which immunotherapy acts. This section sets out how that gap arises and why it bears on the checkpoint response prediction.

The terminology needs care, because the models compared here do not all satisfy the same definition. Where a formal definition is given, two properties recur: organoids arise from stem or progenitor cells capable of self-renewal, and they self-organize into units reproducing part of the architecture and function of the tissue of origin [11,25,26]. No standardized definition has been agreed upon, and the engineering literature instead defines a successful derivation operationally, as the ability to expand, passage, and cryopreserve cells retaining the genetic and histological features of the parent tumor [27].

Measured against those criteria, the models below do not fall on one side of a line. Patient-derived tumor organoids (PDTOs) satisfy both, self-renewing across passages and self-organizing into structures resembling the parent tissue; assembloids are built from them by adding stromal, vascular, or immune populations; spheroids satisfy neither strictly. Air–liquid interface (ALI) cultures resist the label the most, since an undissociated fragment is not established from stem cells and does not self-organize de novo but retains an architecture it never lost, and is generally not expandable. On a strict reading, it is better described as a tissue-fragment-based explant culture, a distinction drawn in the defining literature [26], and is so designated in Table 1 and below.

**Table 1:** Construction strategies for immune-competent tumor organoids: derivation principle, immune and stromal components, strengths, limitations, and representative studies.

Construction Strategy	Derivation/Assembly Principle	Immune and Stromal Components (Retained or Added)	Key Strengths	Key Limitations	Tumor Types Demonstrated	Representative Studies [Ref.]
ALI preservation (endogenous immunity retained)	Undissociated tumor fragments cultured at an air–liquid interface (inner collagen gel, upper surface exposed to air)	Retained: CD8^+^/CD4^+^ T, B, NK cells, macrophages, CAFs, and the native TCR repertoire	Most authentic; preserves in situ spatial architecture and endogenous antigen specificity	Survival limited to days–weeks; not expandable; composition varies with sampling; immune quantity/specificity cannot be titrated	Colorectal, head and neck, clear-cell renal, pancreatic, lung	[15,19,28,29,30,31]
Cluster-based preservation (endogenous immunity retained)	Tumor-like cell clusters prepared under Matrigel-free, serum-free conditions without full dissociation	Retained: tumor-infiltrating lymphocytes, with tumor phenotype and genotype preserved	Very high establishment rate from minimal tissue (reported > 97% from ~10 mg); scales to high-throughput compound screening; lowest tissue input requirement of the preservation formats	Checkpoint-relevant immunological characterization thinner than ALI; published predictive validation concerns chemotherapy response, not ICB; in situ spatial architecture not characterized to the depth reported for ALI	Oral squamous cell carcinoma	[32]
Co-culture: autologous effectors	Expanded epithelial organoids combined with autologous peripheral blood lymphocytes (PBLs) or tumor-infiltrating lymphocytes (TILs)	Added: autologous PBL/TIL; regenerates tumor-reactive T cells with recoverable TCRs	Patient-specific antigen matching; supports killing and IFN-γ readouts	Requires paired blood/tumor material; limited throughput	Colorectal, lung, pancreatic (murine)	[16,17,33,34]
Co-culture: renewable/engineered effectors	Epithelial organoids combined with off-the-shelf engineered effectors	Added: iPSC-NK, CAR-T, CAR-NK, TCR-T	Off-the-shelf; no donor variability; enables potency and on-target/off-tumor toxicity assessment	Allogeneic/synthetic recognition no longer mirrors the patient’s own immune repertoire	Colorectal, breast, hepatocellular, endometrial	[35,36,37,38,39,40,41,42]
Microenvironment reconstruction/assembloid	Directed addition of CAFs/myeloid cells/Treg, or assembly of higher-order assembloids	Added: CAFs, myeloid cells, de-novo-induced Treg	Reconstructs suppressive circuits; models the immunosuppressive tone	Components and geometry must be defined manually; increased complexity	Colorectal, esophageal, pancreatic, breast, glioblastoma	[43,44,45,46,47,48,49,50]
Microfluidic/on-chip refinement	Fragments/spheroids placed in perfused microfluidic chips, or standardized microdissection	Retained (source-derived immune/stromal cells; no defined addition); immune cells retained without dissociation, sustained under perfusion	Prolongs immune function; increases throughput and reproducibility; on-chip response tracks in vivo efficacy; perfusion extends functional immune survival beyond the static ALI window and enables controlled, reproducible fragment size; on-chip PD-1 response has been related to subsequent in vivo efficacy	Does not change the underlying construction principle; still limited by tissue source; requires instrumentation and specialist fabrication; least widely replicated of the preservation formats	Colorectal, lung, hepatocellular, pan-cancer	[51,52,53,54,55]

How to read this table: each row is one design choice, and the columns to its right are its consequences. Those consequences are the immune and stromal material the model contains, what it can and cannot therefore report, and where it has been demonstrated. Reading across a row gives the cost of a design decision; reading down the limitations column identifies which constraint would bind a given study. The operating parameters that representative studies report for each strategy are given in Table 2, and Section 9 maps the same strategies onto what is and is not yet achievable. Abbreviations: ALI, air–liquid interface; CAF, cancer-associated fibroblast; CAR, chimeric antigen receptor; ICB, immune checkpoint blockade; IFN-γ, interferon-γ; iPSC, induced pluripotent stem cell; NK, natural killer; PBL, peripheral blood lymphocyte; PD-1, programmed cell death protein 1; TCR, T-cell receptor; TIL, tumor-infiltrating lymphocyte; Treg, regulatory T cell.

**Table 2:** Reported operating parameters for representative immune-competent tumor organoid protocols, organized by construction strategy.

Construction Strategy (Representative Study)	Starting Material and Tissue Input	Format-Defining Physical Parameter	Immune Input and Effector-to-Target Ratio	Medium and Cytokine Supplementation	Culture Duration and Readout Time Point	Primary Functional Readout
ALI preservation [15]	Undissociated fresh tumor fragments; over 100 human biopsies plus syngeneic murine tumors	Fragments embedded in type I collagen in an inner transwell with the upper gel surface exposed to air; fragment size not specified	None added; the endogenous infiltrate is carried over, so the ratio is inherited from the tissue rather than set	IL-2 optional: T cells persisted to day 21–28 without IL-2 and were expanded with IL-2 at 600 or 6000 IU/mL	CD3^+^ content compared at day 0, day 7 and day 30; checkpoint agent applied for 7 days	TIL frequency by flow cytometry; *IFNG*, *GZMB* and *PRF1* transcripts; tumor cell apoptosis; paired scRNA-seq and TCR-seq
ALI preservation, colorectal [19]	Paired fresh tumor and perilesional colon tissue	Mechanically dissociated fragments in type I collagen in a transwell ALI; fragment size and collagen concentration not reported	None added; a PBMC–Matrigel co-culture was run as the comparator arm	—	Immune composition and cytokines read at day 7; nivolumab and 5-fluorouracil readouts after 5 days of exposure; total culture length not reported	CD45^+^ subset flow cytometry and confocal imaging; somatic mutation profiling; local Treg expansion
Standardized fragment microdissection [51]	Murine B16 melanoma, in a device-development study	Blade spacings of 200, 400, and 800 μm; about 90% of 200 μm fragments fell within 50 μm of target versus about 10% for manual mincing; immune viability was insensitive above roughly 400 μm but reduced at roughly 200 μm	None added; endogenous	—	Viability at day 0 versus day 7; processing 4.5 times faster than manual mincing	Per-lineage viability (CD45^+^, CD11b^+^, CD3^+^, CD4^+^, CD8^+^); immune composition; anti-PD-1 response
Cluster-based preservation [32]	Oral squamous cell carcinoma, from as little as 10 mg of tissue; 161 of 165 specimens (over 97%) established	Matrigel-free, serum-free tumor-like cell clusters prepared without full dissociation; cluster size not reported	None added; endogenous TIL retained, so the ratio is not set	Serum-free and Matrigel-free formulation; composition not specified	Over 100 compounds screened within 2 weeks; prospective observational series of 42 patients	Chemosensitivity, agreeing with neoadjuvant chemotherapy outcome in about 90% of cases (34 of 38 evaluable); a chemotherapy endpoint, not ICB
Co-culture with autologous PBL [16,17]	Expanded epithelial PDTO plus autologous peripheral blood lymphocytes; 11 lung biopsy, 13 lung resection, 15 colorectal biopsy, and 25 colorectal resection samples	Organoids released from the matrix 2 days beforehand and dissociated to single cells on the day of co-culture	Autologous PBL restimulated after week 1; organoid-to-lymphocyte and effector-to-target ratios not reported in the published protocol	Organoids pre-stimulated with IFN-γ at 200 ng/mL for 24 h; co-culture supplemented with IL-2 and anti-PD-1 on anti-CD28-coated plates; IL-2 concentration not stated	Two-week expansion co-culture with restimulation after week 1; 72 h killing assay	Intracellular IFN-γ with surface CD107a or CD137; organoid killing as live EpCAM^+^ DAPI^−^ counts normalized to beads; tumor-reactive CD8^+^ populations obtained from roughly 33–50% of samples
Co-culture with engineered effectors [36,40]	Patient-derived colorectal organoids [36]; malignant pleural effusion-derived organoids from four metastatic breast cancer patients [40]	Three-dimensional organoids in matrix; effector recruitment scored within a 50 μm perimeter zone around each organoid [36]	Ratios of 1:1 to 5:1 across experiments [36]; 1:1 for patient organoids and 2:1 for cell lines [40]	—	8 h luciferase cytotoxicity endpoint and 10 h live imaging, with 48 h before replating [36]; 72 h with readouts at 0, 24, 48, and 72 h [40]	Luciferase-based target viability; loss of fluorescent organoid area; effector recruitment density; antigen-matched lysis across CD276, HER2, EGFR, TROP2 and EpCAM
Microenvironment reconstruction [49]	Colorectal tumor organoids, two murine and two human lines, plus naive CD4^+^ T cells	Transwell co-culture, so the interaction is indirect	Organoid-to-T-cell ratio not reported; the suppression assay used induced Treg and peripheral blood mononuclear cells at 1:1	No exogenous TGF-β concentration reported; TGF-β dependence probed with SB-431542 at 10 μM and LY364947 at 1 μM; sodium L-lactate 10 mM, GSK2837808A 10 μM, and dichloroacetate 5 mM	2 and 5 days, with 5 days used throughout, and a separate 4-day suppression assay	Proportion of CD4^+^CD25^+^FOXP3^+^ cells; RNA-sequencing Treg signature; association of that signature with survival
Perfused microfluidic chip [53]	Dissociated tumor cells infused into isolated microwell arrays; murine orthotopic breast tumors differing in PD-L1 level, plus primary human breast and renal tissue	Up to 960 mini-tumors per chip across four parallel injection ports	None added; immune cells are carried in with the dissociated suspension, so the ratio is not set	—	Anti-PD-1 response resolved within 36 h; on-chip interrogation at day 10 after inoculation against an in vivo endpoint at day 24; antibody concentration not reported	Time-lapse live-cell imaging of immune–tumor dynamics; viability; IFN-γ, TNF-α, and IL-6 secretion
Suspension in ultra-low-attachment plates [56]	Peripheral blood-derived chronic lymphocytic leukemia cells; a non-solid-tumor model, included here because it calibrates the effector ratio to tissue	Ultra-low-attachment plates against round-bottom 96-well two-dimensional controls; follicle-like structures form over time	Tumor-to-T-cell ratios set to match those measured in lymph node rather than blood samples; the numeric ratio is not given, and samples were selected at above 10% CD4^+^ T cells from paired blood and lymph node comparison in 24 treatment-naive patients	—	Expansion sustained for up to 7 weeks; drug assays over 4–6 days	T-cell-dependent proliferation and survival; spatial localization; BTK-inhibitor and venetoclax response

Every parameter is reported as stated in the cited source. An em dash denotes a parameter that the source does not report; no value has been estimated or inferred where a source is silent. The pattern of omissions is itself informative: effector-to-target ratio, cytokine concentration, and total culture duration are the parameters most often left unstated, which is the reporting deficit underlying the standardization problem taken up in Section 9. Where a study used a non-human or non-solid-tumor model, this is stated in the second column, and the parameters should be read as characterizing the format rather than as validated human protocols. Abbreviations: ALI, air–liquid interface; BTK, Bruton tyrosine kinase; CD276, B7-H3; DAPI, 4′,6-diamidino-2-phenylindole; EGFR, epidermal growth factor receptor; EpCAM, epithelial cell adhesion molecule; *GZMB*, granzyme B (gene); HER2, human epidermal growth factor receptor 2; ICB, immune checkpoint blockade; IFN-γ, interferon-γ; *IFNG*, interferon-γ (gene); IL, interleukin; PBL, peripheral blood lymphocyte; PBMC, peripheral blood mononuclear cell; PD-1, programmed cell death protein 1; PD-L1, programmed death-ligand 1; PDTO, patient-derived tumor organoid; *PRF1*, perforin 1 (gene); scRNA-seq, single-cell RNA sequencing; TCR-seq, T-cell receptor sequencing; TGF-β, transforming growth factor-β; TIL, tumor-infiltrating lymphocyte; TNF-α, tumor necrosis factor-α; Treg, regulatory T cell; TROP2, trophoblast cell surface antigen 2. Retaining the word organoid for the whole group requires justification. It is used here as an umbrella term for patient-derived, three-dimensional, immune-competent tumor models, in keeping with the prevailing usage and because these models share the property that this review concerns: they preserve or reconstruct a functional tumor–immune interaction in patient-derived material. The umbrella carries no claim that every model meets a common developmental definition; where the distinction bears on the interpretation, the specific format is named, and inferences resting on self-renewal or long-term expansion apply to organoid-proper formats rather than explant cultures.

Two derivation routes underlie these formats. The submerged culture embeds the dissociated epithelium in the extracellular matrix, whereas the ALI culture maintains undissociated fragments with direct access to air. Whether immune cells survive derivation at all depends largely on that choice.

As a model of the epithelial compartment, organoid technology reaches a fidelity that older systems rarely match. Living biobanks from colorectal and breast cancers reproduce the histology, driver mutation spectrum, and transcriptional subtype of the parental lesions, and they stay stable across passages. Structured colorectal libraries also retain rare subtypes and the progressive loss of the niche factor dependence that marks malignant progression [12,13,57]. This fidelity carries into function across tumor types [11,14,58,59].

The range of culture formats also matters, since the format is itself a construction variable. The first format, the scaffold-free suspension culture, dispenses with an exogenous matrix. The matrix-free prostate organoids retained patient-specific lineages and active androgen receptor signaling, whereas the Matrigel-grown counterparts drifted toward basal-like states through laminin-binding integrin engagement [60]. The suspension also supports the immune co-culture, sustaining the T-cell-dependent proliferation for several weeks at effector ratios matched to the lymph node tissue [56]. What it gives up is structural support for invasive growth.

Functionalized hydrogels take the opposite approach, replacing an undefined biological matrix with a chemically defined one whose composition and mechanics can be set. A synthetic gel built around the laminin–integrin cue that pancreatic organoids require supported both normal and malignant growth and allowed stromal co-embedding, showing that a defined gel can replace Matrigel outright [61]. Such matrices also make matrix properties experimental: in gels mechanically matched to pancreatic tumors, organoids became resistant to several chemotherapies through the stiffness-driven, CD44-hyaluronan-dependent drug efflux, reversing in a softer gel [62]. Neither study included immune cells, so the consequences of the matrix design for immune function are inferred rather than demonstrated, and we are not aware of a defined-hydrogel platform relating matrix mechanics directly to PD-L1 expression or T-cell cytotoxicity.

The trade-off runs the same way across these formats. Structural maintenance and microenvironmental recapitulation are the highest where the architecture is never disrupted and stromal and immune cells are carried over intact, intermediate in matrix-embedded and hydrogel systems, and the lowest in suspension. Application follows, and this review concentrates on the formats in which immune competence has actually been achieved: fragment-based preservation and matrix-embedded co-culture.

That success rests on the one compartment that immunotherapy cannot ignore. The checkpoint blockade acts on the signaling among malignant cells, T cells, and myeloid and stromal populations, none of which a purified epithelial culture contains. Two-dimensional cell lines have drifted clonally and carry no host immunity, and patient-derived xenografts keep their architecture but grow in immunodeficient hosts [10]. A standard PDTO is much the same, because dissociation and epithelium-selective conditions strip out infiltrating lymphocytes and antigen-presenting cells within a few passages [11].

**Figure 1:**
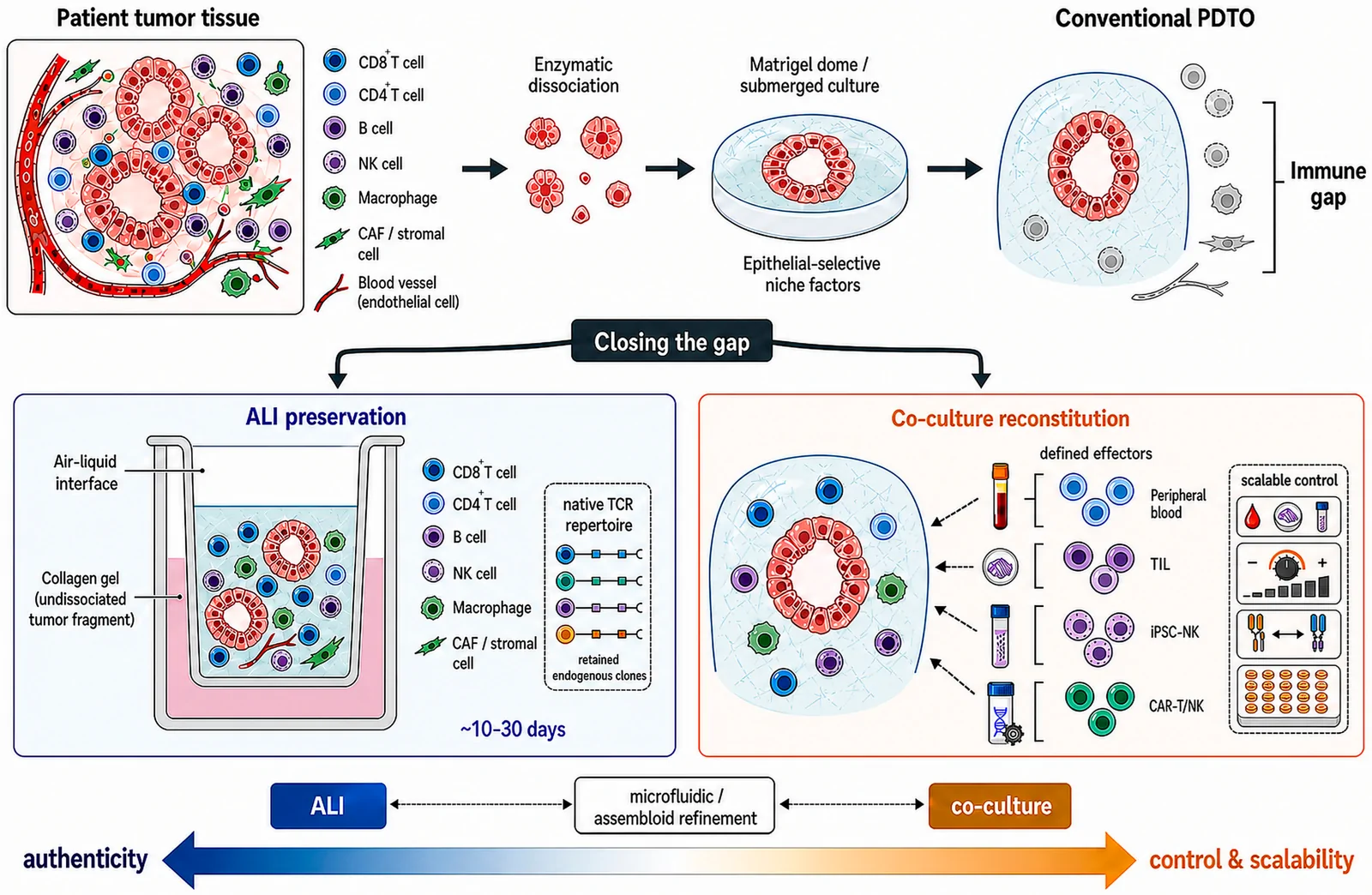
The immune gap in conventional tumor organoids and the two strategies that close it: preserving endogenous immunity versus reconstituting it by co-culture. Enzymatic dissociation, extracellular matrix embedding, and epithelium-selective niche factors strip conventional patient-derived tumor organoids (PDTOs) of tumor-infiltrating lymphocytes (TILs), myeloid cells, cancer-associated fibroblasts (CAFs), and vasculature within a few passages, leaving the “immune gap.” Strategy I, shown as ALI preservation, propagates undissociated fragments at an air–liquid interface (ALI). It retains CD8^+^ and CD4^+^ T cells, B cells, natural killer (NK) cells, macrophages, and CAFs, together with the native T-cell receptor (TCR) repertoire. These remain functional for roughly 10–30 days [15,19,31]. Strategy II, shown as co-culture reconstitution, adds defined effectors to expanded epithelial organoids as a controlled, scalable variable: autologous peripheral blood lymphocytes (PBLs), TILs, iPSC-derived NK cells, or engineered CAR-T/CAR-NK cells [16,17]. The branches sit at opposite ends of a spectrum running from authenticity to control and scalability, bridged by microfluidic and assembloid refinements. This dichotomy, not tumor type, organizes the review. ALI, air–liquid interface; CAF, cancer-associated fibroblast; CAR, chimeric antigen receptor; iPSC, induced pluripotent stem cell; NK, natural killer; PBL, peripheral blood lymphocyte; PDTO, patient-derived tumor organoid; TCR, T-cell receptor; TIL, tumor-infiltrating lymphocyte.

A model that cannot present tumor antigen, engage a T-cell receptor, or maintain a suppressive myeloid niche cannot say whether a patient’s tumor will respond to the PD-1 blockade, however faithfully it copies the epithelial genotype. Two strategies have grown up to close the gap: one preserves the endogenous infiltrate during derivation, while the other reconstitutes immunity by adding defined effectors. Both are feasible, so the question is which better rebuilds the interactions that set the checkpoint sensitivity, and how durably (Figure 1). Because the boundary can be read in more than one way, the criterion used here is stated explicitly.

The distinction is drawn on a single primary criterion: whether the immune compartment is separated from the tumor tissue during derivation and then reintroduced. In preservation, no dissociation step intervenes, so the immune and stromal cell number, phenotype, and position are inherited from the tissue rather than set by the experimenter. In reconstitution, immune cells enter as a separately prepared input, and their identity, dose, and geometry become experimental variables.

Two features that might seem decisive are not used as the criterion. The first is the source of the immune cells. The co-culture with tumor-infiltrating lymphocytes isolated from the same tumor is classified here as reconstitution, because those lymphocytes were separated and then re-added. Their original frequency and spatial arrangement did not survive, and the effector-to-target ratio is whatever the investigator chose. The autologous origin confers patient-specific antigen matching, but that is a property of the immune cell source rather than the construction route and is treated as an independent axis. The second is spatial fidelity, which follows from the primary criterion rather than defining it and varies continuously within each route; it is therefore a descriptive attribute in Table 1. Applied consistently, the criterion sorts the models cleanly: fragment-based cultures preserve, whereas every co-culture format reconstitutes.

An indirect co-culture falls on the reconstitution side under the same criterion, since immune cells separated by a transwell insert or conditioned medium remain a separately prepared input. Removing direct contact makes these a useful special case, because it isolates the soluble factor crosstalk from contact-dependent killing. The conditioned medium from activated natural killer cells carries soluble death ligands; yet, it failed to kill colorectal organoids that the contact-dependent co-culture killed efficiently. That result locates the cytotoxic mechanism in direct engagement [63].

## 4. Construction Strategies I: Preserving Endogenous Immunity

The first strategy does not add immune cells at all; it keeps them from being removed. The air–liquid interface (ALI) culture propagates a tumor as an intact fragment rather than dissociated epithelium, carrying the endogenous infiltrate, stromal cells, and native antigen receptor repertoire into the dish. Containing immune cells is not enough; what counts is how completely and how durably such a model rebuilds the interactions governing the checkpoint response. Table 1 compares the six construction strategies attribute by attribute, and Table 2 the operating parameters representative studies report, together with those they leave unstated.

Once the tissue architecture is enzymatically broken, it cannot be rebuilt, whereas an undissociated fragment keeps it by default. In the original method, fragments were set in a collagen gel within an inner transwell whose top surface met the air. This gives dense tissue the oxygenation it needs, while epithelial, immune, and stromal elements hold their original spatial relationships. Single-cell RNA sequencing with paired T-cell receptor sequencing showed that infiltrating CD8^+^ and CD4^+^ T cells, B cells, natural killer (NK) cells, and macrophages were all retained. The receptor repertoire also matched the originating tumor rather than a culture-selected subset [15]. That separates preservation from mere co-presence: the antigen specificities the tumor had already raised are carried forward. Under the PD-1 or PD-L1 blockade, these organoids expanded the tumor-antigen-specific T cells that killed their targets, confirming that an endogenous, functional checkpoint axis had survived derivation.

Preservation-based construction now spans many tumor types, turning ALI from a single landmark into a general strategy. Colorectal ALI organoids keep αβ and γδ T cells, B cells, and innate lymphoid and myeloid populations alongside matched somatic mutations, revealing adaptive behaviors that dissociated systems miss [19]. Head and neck tumors retain tumor-infiltrating lymphocytes beside cancer-associated fibroblasts, with pembrolizumab drawing out more interferon-γ and a shifted CD8^+^/CD4^+^ ratio [28]. Clear-cell renal organoids hold the parental histology, immune cells, and molecular profile, and respond variably to nivolumab [29]. Pancreatic cultures retain myofibroblastic, inflammatory, and antigen-presenting fibroblast subsets beside CD8^+^ T cells and macrophages [30]. Bladder organoids keep immune subsets at the early passage and support drug screening [64]. Lung cancer shows the central constraint most clearly: an optimized protocol extended immune cell survival from roughly ten days to 30, with single-cell sequencing confirming the persistence of CD45^+^ immune cells and α-SMA^+^ fibroblasts [31]. That extension had to be engineered: the more authentic the model is, the less stable it tends to be.

Because the viability window is short, several engineering fixes have targeted the reproducibility and lifespan of the ALI fragment. The fragment size matters for both the immune content and anti-PD-1 responsiveness, with the viability holding above roughly 400 μm but dropping below 200 μm. Hand-mincing therefore introduces uncontrolled variation, whereas purpose-built microdissection tools cut uniform fragments several times faster and keep the immune composition and checkpoint responses intact [51]. Sustaining immune function under continuous flow is the other target, and the microfluidic formats developed for it [52,53,54] are taken up in the following subsection on microfluidic and organ-on-chip refinement.

The ALI culture is not the only route to a preserved immune compartment. Patient-derived tumor-like cell clusters are prepared under Matrigel-free, serum-free conditions retaining tumor-infiltrating lymphocytes with tumor phenotype and genotype. In oral squamous cell carcinoma, the approach established cultures from over 97 percent of specimens, using as little as 10 mg of tissue. It also screened more than 100 compounds within two weeks. Sensitivity testing agreed with the clinical outcome in roughly 90 percent of evaluable cases, drawn from a prospective series of 42 patients [32]. Two qualifications are essential. The clinical endpoint was a response to neoadjuvant chemotherapy, not the checkpoint blockade, so the concordance figure speaks to chemosensitivity prediction and carries no implication for ICB prediction, which remains untested in this format. And the minimal tissue requirement addresses the input tissue constraint that limits the ALI culture to generous surgical specimens. The construction principle is still preservation.

The evidence supporting each strategy differs in kind, so Table 1 compares them attribute by attribute; the subsection that follows takes up the microfluidic formats and the question of the relative translational potential in detail.

### Microfluidic and Organ-on-Chip Refinement of Preservation-Based Models

Microfluidic and organ-on-chip formats are treated separately here because they address transport, the constraint that most limits preservation-based models.

A preserved fragment is dense tissue without a circulation, so oxygen and nutrients reach its interior only by diffusion. The air–liquid interface answers the oxygenation half by exposing the gel surface to air [15]. It does so statically, however, without delivering nutrients or clearing waste. That is the main reason endogenous immune cells in a static culture persist only days to a few weeks. Continuous perfusion makes the culture duration an engineerable parameter.

Of the three architectures pursued, the perfused device culture of organotypic spheroids retains autologous lymphoid and myeloid cells. It also reproduces both the sensitivity and resistance to the PD-1 blockade, with distinguishing cytokine signatures. A checkpoint response can therefore be read on-chip in material that carries its own immune compartment [52]. Massively parallel formats address the throughput: mini-tumor chips generate hundreds of microtumors from one specimen, and the on-chip anti-PD-1 responses at day 10 corresponded to the later in vivo efficacy [53]. Supported-chip designs instead pursue establishment reliability, using mesenchymal stromal cells to raise success rates and improve how accurately PD-L1-blockade responses are reproduced [54]. A droplet-based variant bridges formats, micro-organospheres retaining native stromal and immune cells while permitting rapid, scalable profiling [55]. The standardized microdissection belongs alongside these, since a uniform fragment size is a precondition for reproducible device loading [51].

Whether these systems carry a greater translational potential than the ALI culture is best answered by the dimension. Perfused formats have the advantage on the throughput, reproducibility of fragment handling, and duration of functional immune survival, and provide the only preservation-format readout linked to a subsequent in vivo outcome [53], though a single linkage is not a validated predictor. The ALI culture remains the reference for the depth of the immunological characterization [15,19] and for the in situ spatial fidelity, since loading a fragment into a device does not restore the architecture the preparation step did not preserve. A claim of overall superiority is not supported in either direction.

The devices require fabrication and instrumentation that most tumor biology laboratories do not hold, which is why these formats are the least replicated of the preservation strategies, and the device geometry adds a further source of inter-laboratory variation. Perfusion does not create a vasculature: the medium passes through channels adjacent to the tissue rather than a perfused endothelial network, so the model still lacks endothelial barrier function, immune cell extravasation, and realistic drug distribution. Consistent with the criterion of Section 3, perfusion changes the conditions under which a preserved compartment is maintained but not the principle by which it was obtained. That is why these formats are treated as a refinement of the preservation route rather than a third strategy.

The translational draw of preservation is that these are arguably the most native immune-competent models available. The immune cells are the patient’s own, and they enter culture in the proportions the tissue had. That authenticity is what allowed colorectal ALI organoids to show a local regulatory T-cell expansion after the PD-1 blockade, where matrix-assembled peripheral blood co-cultures did not [19]. The same design limits it: a finite infiltrate fades over days to a few weeks unless actively maintained [31], and neither the antigen specificity nor immune cell number can be dialed up or down. Preservation buys fidelity at the cost of control and durability.

Fidelity claims need a stricter test than detection. Single-cell and repertoire data establish that T, B, natural killer, and myeloid cells remain present, and that the receptor repertoire matches the parent tumor [15]. The presence and repertoire identity are necessary but not sufficient. A model could retain every lineage while shifting the relative frequencies, narrowing the clonal diversity, or driving cells toward an exhausted state, and still be scored immune-competent. The reported viability windows describe how long immune cells can be detected [31], not how long they remain representative. What would settle this are paired measurements of the composition, clonal structure, and functional state between fresh tissue and the cultured model at defined time points, reported as a change rather than an endpoint. Such data remain sparse, so the drift should be treated as an open variable.

Culture conditions are the second reason immune competence cannot be attributed to the derivation technique alone. Media built around epithelial niche requirements act on more than the epithelium. The growth factor composition shapes the tumor cell differentiation state and can alter the surface phenotype, with PD-L1 among the relevant markers. The cytokine supplementation intended to keep lymphocytes alive also sets their activation state. The matrix stiffness feeds back on the tumor phenotype independently of any immune manipulation [61,62]. A checkpoint readout obtained under one formulation is therefore conditioned on that formulation. This does not weaken the argument that construction determines prediction; it specifies what construction has to mean. Construction is not only the derivation route but the full set of choices defining the model: the medium formulation and niche factors, cytokine support, matrix, culture duration, and readout time point. Two laboratories using the same nominal route and different media are running different constructions, so reporting these parameters explicitly is a precondition for comparing the results, and is one of the standardization requirements returned to in Section 9.

## 5. Construction Strategies II: Reconstituting Immunity by Co-Culture

Where preservation inherits an authentic but short-lived infiltrate, the second strategy builds immunity from defined, renewable parts. An epithelial organoid is expanded to the numbers screening and biobanking need, and immune effectors are then added as a controlled variable: autologous lymphocytes, tumor-infiltrating cells, or engineered T and natural killer cells. This trades the native spatial context of the ALI culture for control over which immune cells are present, how many, and with what specificity.

A patient’s peripheral blood holds tumor-reactive T cells that an autologous organoid can selectively expand. Co-cultured with matched organoids from mismatch-repair-deficient colorectal and non-small-cell lung cancers, peripheral blood lymphocytes enriched CD8^+^ T cells that recognized and killed the autologous tumor while sparing healthy organoids from the same patient, without prior knowledge of the target antigen [16]. The two-week protocol was later pinned down, with a set cytokine milieu, defined organoid-to-lymphocyte ratios, and readouts of degranulation, interferon-γ release, and organoid killing [17]. Specificity here is generated in the culture.

The effector source sets a spectrum from autologous authenticity to renewable standardization. At the autologous end, repeatedly restimulated tumor-infiltrating lymphocytes grow into organoid-reactive populations and yield tumor-specific T-cell receptors directly [33], and, in lung cancer, expanded lymphocytes killed the matched organoids, with the profiling separating the proliferative-to-effector-memory programs accompanying cytotoxicity [65]. At the far end, induced pluripotent stem cell (iPSC)-derived natural killer cells stay highly cytotoxic and, with activated T cells and anti-PD-1, tighten the tumor control without donor variation [35]. Between the poles, allogeneic and cross-species systems isolate the mechanisms: murine pancreatic co-cultures confirm the principle is species-independent [34], and the conditioned medium from activated NK cells failed to kill colorectal organoids, locating the cytotoxicity in contact-dependent engagement [63].

The co-culture is also the standard functional assay for engineered effectors, with a patient-derived organoid as an antigen-defined target. Chimeric antigen receptor (CAR)-engineered NK cells aimed at a tumor-specific neoantigen achieved near-complete selective killing in a colorectal model. A broadly expressed antigen, by contrast, spared neither the tumor nor normal epithelium. That contrast was an early sign that organoids report the efficacy and on-target off-tumor toxicity at once [36]. That dual readout has spread across effector classes and tumor types. B7-H3-directed CAR-T cells gave a dose-dependent killing that carried through to xenografts [37]. T cells armed against a shared β-catenin driver mutation eliminated colorectal and endometrial organoids [38], and combined MICA/B and NKG2A targeting exposed the innate-axis vulnerabilities [39]. Adapter CAR-T cells against metastatic breast organoids killed in proportion to antigen expression across HER2, TROP2, and EpCAM [40], and hepatocellular organoids modeled glypican-3–directed responses [41]. Imaging pushes these endpoints to single-cell behavior [42].

Reconstitution delivers control, scale, and a natural fit with engineering, at an interpretive cost, since immunity added to an epithelial organoid does not rebuild the original spatial arrangement. Two consequences bear on prediction. Quantitatively, effector-to-target ratios are set to give a measurable signal within a short assay. They are not calibrated to the immune cell density of the patient tumor, and they can considerably exceed the intratumoral proportion. That gap is widest in the immune-poor tumors where a predictive assay would be most valuable. A response measured under a supraphysiological effector load may therefore overstate what the tumor could mount in situ. Spatially, immune cells concentrate into localized niches, and coordinated cellular neighborhoods, rather than overall densities, govern antitumoral immunity [66]. Dispersed effectors reproduce an average exposure, and they reproduce neither the enriched neighborhoods where the checkpoint blockade acts in vivo nor the excluded regions behind much clinical resistance. The co-culture therefore tends to model a uniformly infiltrated tumor, which is the minority case clinically. Autologous formats preserve patient-specific antigen matching but need paired material and stay low in throughput, whereas renewable and engineered sources introduce recognition no longer mirroring the patient’s repertoire. Such models answer the questions of effector potency and antigen specificity more precisely than ALI, but construction alone cannot indicate whether a reconstituted response would survive an intact suppressive niche.

## 6. Rebuilding and Reading out the Tumor Immune Microenvironment

**Figure 2:**
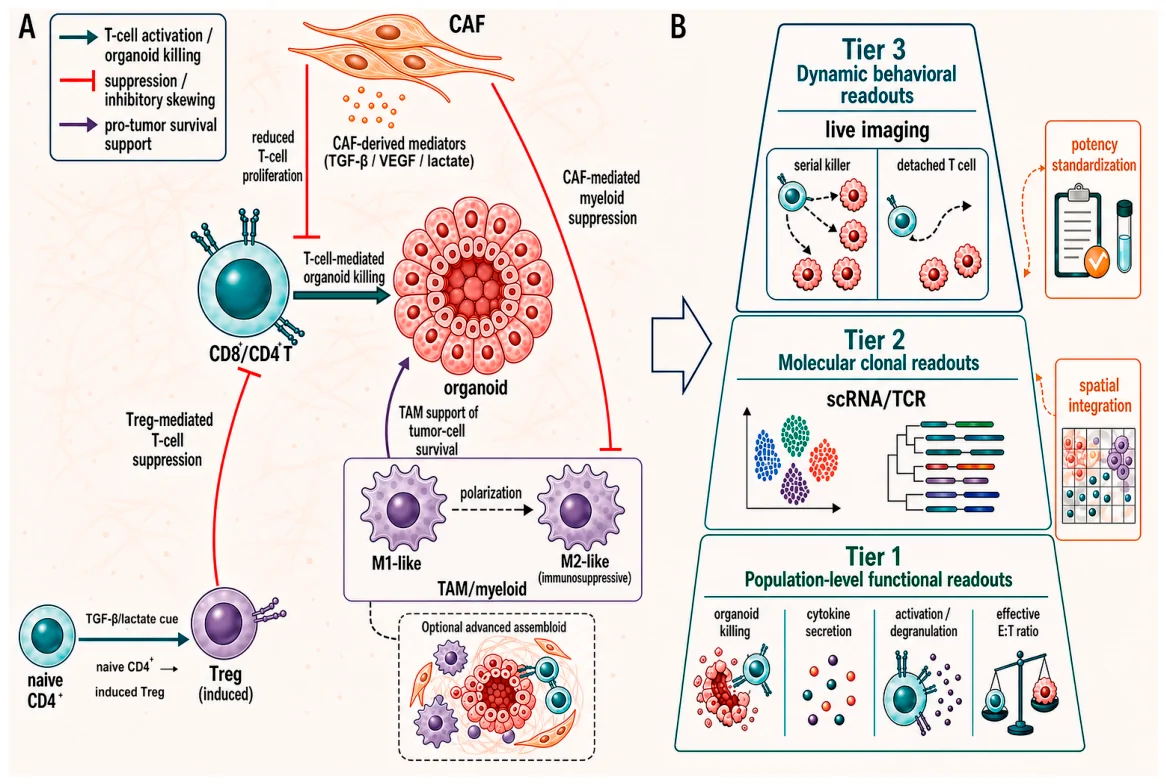
Reconstructing the suppressive tumor immune microenvironment in organoid co-cultures, and the readout hierarchy used to interrogate it. The two are coupled: a reintroduced suppressive population is informative only if its influence can be measured, and a detailed readout only if the microenvironment is complete. (**A**) Defined suppressive populations are added around a central organoid–effector interface. CAFs secrete transforming growth factor-β (TGF-β), vascular endothelial growth factor (VEGF), and lactate. These restrain T-cell proliferation and steer monocytes toward an immunosuppressive phenotype. Chemotherapy-induced immunogenic signals can override that phenotype by repolarizing macrophages toward a pro-inflammatory, phagocytic state [43,44,46]. Comparable tumor–fibroblast assembloids exist for esophageal adenocarcinoma [45]. Tumor-associated macrophage subsets are resolved in pancreatic and breast organoid systems and linked to cancer cell survival [47,48]. Regulatory T cells (Treg) are induced de novo when colorectal organoids drive naive CD4^+^ T cells toward a suppressive phenotype under TGF-β and lactate, with the induced signature corresponding to shorter survival [49]. A glioblastoma tumor–immune organoid reproduces antigen-specific, checkpoint-responsive T-cell dynamics within organized tissue [50]. Arrows are color-coded by interaction type, following the key in panel A: teal arrowheads for T-cell activation and organoid killing, red blunt ends for suppression and inhibitory skewing, and purple arrowheads for pro-tumor survival support. Dashed arrows mark differentiation or polarization steps. (**B**) Readouts, with resolution increasing upward. At the population level (Tier 1), these are organoid killing, interferon-γ (IFN-γ) and tumor necrosis factor-α (TNF-α) secretion, CD137/CD107a expression, and effective effector-to-target (E:T) ratios. Fragment reactivation capacity tracking clinical outcome belongs here too [67,68]. At the molecular-clonal level (Tier 2), single-cell RNA sequencing (scRNA-seq) paired with T-cell receptor (TCR) sequencing resolves clonal expansion and transcriptional-state shifts under checkpoint blockade [15]. At the dynamic-behavior level (Tier 3), high-content imaging separates serial-killer from disengaged T cells and defines their transcriptional signatures [42]. Two gaps are marked: no shared potency metric [69], and spatial omics not yet integrated in immune-competent organoids. CAF, cancer-associated fibroblast; E:T, effector-to-target; IFN-γ, interferon-γ; scRNA-seq, single-cell RNA sequencing; TAM, tumor-associated macrophage; TCR, T-cell receptor; TGF-β, transforming growth factor-β; TNF-α, tumor necrosis factor-α; Treg, regulatory T cell; VEGF, vascular endothelial growth factor.

Two-component organoid–effector co-culture measures whether immune cells can kill tumor cells. The checkpoint response, however, is part of a larger circuit in which fibroblasts, regulatory T cells, and myeloid cells actively suppress killing. Moving toward a model of checkpoint biology therefore demands two advances together: rebuilding the suppressive cell types a two-component system omits, and establishing readouts that gauge how strongly those cells bend the antitumor response (Figure 2).

Rebuilding the microenvironment starts with the stromal and regulatory populations that turn a killing assay into a model of immune suppression. Reviews of stroma-targeted modeling note that the non-malignant stromal composition is what conventional systems reproduce least well. The same reviews identify the co-culture with patient-derived organoids as the principal route to rebuilding it [70]. Cancer-associated fibroblasts are the most consequential additions, imposing the immunosuppressive tone of mesenchymal-subtype tumors and skewing monocytes in a way that chemotherapy-induced immunogenic signals can reverse, so the niche is changeable rather than fixed [43,44,45,46]. Myeloid diversity, de novo regulatory T-cell induction, and higher-order tumor–immune assembloids extend the same principle to the remaining suppressive compartments (Figure 2A) [47,48,49,50].

A rebuilt microenvironment is only useful through readouts that quantify immune function, and these span a hierarchy from bulk effect to single-cell behavior (Figure 2B). At the base, organoid killing, interferon-γ secretion, and activation markers measure the effector engagement, as in a head and neck platform that also defined the effective effector-to-target ratios [67]; an intact fragment’s reactivation capacity under the PD-1 blockade tracked the clinical outcome [68]. Above it, paired single-cell and T-cell receptor sequencing shows which clones expand under the checkpoint blockade [15], and high-content imaging adds the behavioral read that endpoint assays lack [42].

What they cannot do is compare across laboratories, the first of two gaps blocking the step to prediction. Cell-therapy manufacturing makes the contrast explicit: there potency assays are tied to a mechanism of action [71], and validated flow-cytometric killing assays for CAR-T release meet the analytical bar that standardization demands [72]. Organoid readouts have nothing of the kind, quantifying killing by mismatched imaging pipelines and effector-to-target ratios that vary between groups [73,74], so the results cannot be pooled toward a single predictive threshold [69]. Fixing this means agreeing on what makes the results comparable: killing normalized to a defined effector-to-target ratio, activity referenced to a shared benchmark, and endpoints tied to a stated mechanism of action.

Coupling single-cell and receptor sequencing to spatial profiling inside immune-competent organoids is the second gap, and it is still mostly undone. In intact tumors, the CODEX profiling of the colorectal invasive front showed that coordinated cellular neighborhoods, not densities alone, govern antitumoral immunity [66], and the spatial dissection of the tumor immune landscape has been proposed as a route to precision medicine [75]. Both gaps matter because the reconstructed microenvironment is where suppression blunts or permits an antitumor response.

### Multi-Omic and Computational Layers of the Readout

The readouts described so far are measured one modality at a time, while the decision they inform draws on several. The layered profiling of organoids is already feasible: a liver cancer organoid biobank characterized across genomic, epigenomic, transcriptomic and proteomic layers recovered subtypes that tracked the patient outcome and segregated the drug response patterns [76]. Spatial methods extend the logic without discarding the position, mapping transcript, protein, metabolite and chromatin landscapes across three-dimensional cultures, though fusing the multimodal data and turning the result into a clinically usable biomarker remain acknowledged obstacles [77]. Neither study used an immune-competent system: the machinery exists and is trained on intact tumors [66,75], not on the reconstructed compartment.

Computation is the second layer, and, here, the evidence is thinnest relative to the enthusiasm it attracts. Deep-learning image analysis scores individual organoids from label-free images [78,79], and network models trained on organoid pharmacogenomic data have predicted the patient drug response with external clinical validation [80]. All three concern cytotoxic and targeted agents in epithelial organoids, not the checkpoint blockade in immune-competent ones, and that distinction should not be elided. Digital twins warrant the same caution: currently built from imaging, mechanistic models, and clinical records, with data integration, validation, scalability, and governance unresolved, and no organoid-anchored instance reported [81]. Nearer the term, the role is narrower: computation is how the readouts of Figure 2B become one comparable quantity, which is what the missing potency standard requires.

## 7. Application: Predicting Immune Checkpoint Blockade Response

Building an immune-competent organoid serves one clinical question: will this patient’s tumor respond to the checkpoint blockade? The evidence orders itself by the predictive capability tested rather than by the cancer type, and Table 3 sorts and grades it on four such capabilities: registering a checkpoint effect, capturing the response biology that static biomarkers miss, reproducing the resistance, and aligning with real-world response rates. Across all four, the evidence reads as an emerging proof-of-concept from small, largely retrospective cohorts, drawn predominantly from colorectal cancer with supporting observations in the head and neck, gastric, renal, and lung cohorts. Breast cancer and most other solid tumors remain at an earlier, largely methodological phase.

The first requirement is to register a checkpoint-dependent functional response, which several platforms now manage dose-dependently. Organoids retaining tumor-infiltrating lymphocytes shrank dose-dependently under nivolumab, with reactivity in both PD-L1-positive and PD-L1-negative tumors [18], building on the feasibility work in matched melanoma and lymph node tissue [82]. The throughput has since climbed: micro-organospheres register checkpoint-induced killing on a clinically compatible timescale [55], and colorectal microtumors mounted measurable interferon-γ responses to pembrolizumab [83]. Microfluidic formats reproduce the PD-1-blockade sensitivity and resistance while tracking the in vivo efficacy [52,53]. Disrupting the HER2-induced PD-L1 expression in gastric organoids also reduced immune evasion [84]. A checkpoint effect is detectable functionally.

A functional assay becomes clinically interesting only when it captures the response biology that molecular biomarkers cannot. The mismatch repair status is the dominant correlate of the colorectal checkpoint benefit, and organoids follow that axis while adding resolution. Microtumors from microsatellite-unstable tumors mounted strong interferon-γ responses, and stable ones generally did not. One microsatellite-stable, POLE-mutated, high-burden tumor still trended toward a response, an individual result that no single biomarker would have called [83]. An organoid readout can fold several determinants into one functional output, an argument carried through in Section 8.

Prediction must cover failure too, and immune-competent organoids reproduce resistance as well as sensitivity. Colorectal ALI organoids under a PD-1 blockade showed local regulatory T-cell expansion, a correlate of adaptive resistance absent when peripheral blood cells were mixed with organoids in the matrix, indicating only the preservation-based setup kept the responsible suppressive circuitry [19]. Resistance can also be probed molecularly: the caspase-based quantification of T-cell-induced apoptosis identified resistance-associated markers including REG4, whose knockout restored immune sensitivity [85]. Such findings nominate targets to reverse resistance, most of all for the microsatellite-stable majority that the current biomarkers leave out.

A live organoid can also be sampled repeatedly across a treatment course, and the checkpoint response is a trajectory rather than a single state. The immune composition and clonal dynamics shift measurably between the baseline and early on-treatment sampling, in ways that no pretreatment snapshot captures [92]. Because a resected specimen yields a renewable culture, the same tumor can be read before and after ex vivo exposure and manipulated as a patient cannot. The limits are just as concrete. The serial passage of one pretreatment specimen does not generate the immune clonal evolution therapy drives in the patient; the later selection reflects the culture conditions rather than in vivo immune pressure, nor is there a systemic compartment: lymph node priming, the recruitment of peripheral effectors, the systemic sequelae of checkpoint blockade, and the pharmacokinetics all lie outside a closed culture, and a resection specimen represents one site at one time. These constraints press the hardest on preservation-based models, where the infiltrate persists only briefly; reconstituted models can be re-supplied, but each re-supply is an experimental input rather than an evolved population. Serial ex vivo sampling therefore supports the controlled perturbation of a fixed starting state, not a simulation of the patient trajectory.

The hardest test is whether the organoid-derived response rates line up with the clinical outcomes, and the evidence is preliminary and patchy. In head and neck cancer, ALI organoids treated with pembrolizumab amplified tumor-reactive CD8^+^ T cells in a subset of cases [28], but matching such readouts to the response rates remains a near-term goal. Real-world benchmarks set the target—among them, the near-complete pathological responses to the neoadjuvant PD-1 blockade in mismatch-repair-deficient colon cancer [91], while other cohort-scale correlations exist only as conference abstracts and must be weighed as low-level evidence pending peer review. Breast cancer marks the edge of the current knowledge most sharply. The clinical need is pressing, set by the approvals of pembrolizumab and atezolizumab in triple-negative disease and backed by the overall-survival and updated efficacy data from the pivotal trials [3,93,94,95,96,97]. Even so, no peer-reviewed study yet shows a breast organoid directly predicting the checkpoint response. What exists so far is a methodological groundwork of several kinds. Cancer-on-chip systems and patient-derived scaffolds quantify the CAR-T efficacy and microenvironment-driven differences in PD-L1 regulation and T-cell cytotoxicity. Largely non-malignant ductal organoids characterize innate-like T-cell populations, a small series correlated Vδ1^+^ γδ T-cell representation with survival, and stromal models map the tumor–stroma interactions [86,87,88,89,90,98,99]. This is the baseline knowledge of breast-resident immunity, not direct prediction, and that gap is what makes breast cancer a proving ground rather than a success story.

**Figure 3:**
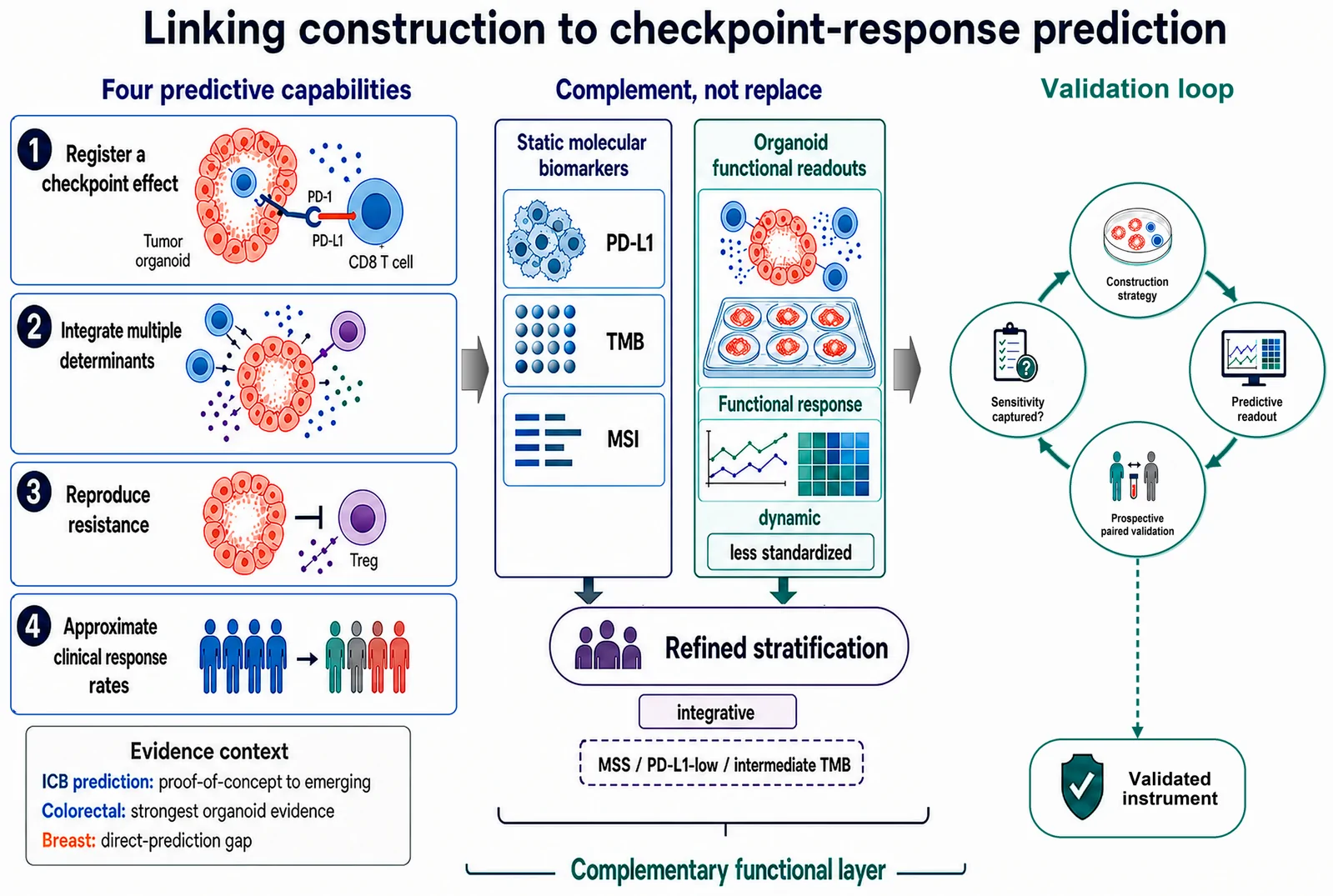
Linking construction to prediction: how construction strategies map onto four predictive capabilities, complement molecular biomarkers, and feed a validation loop. The review’s central argument, that construction determines prediction, resolves into three linked zones. (Left) Four predictive capabilities, each annotated with the construction strategy that best realizes it and its evidence level. ① Register a checkpoint effect: dose-dependent killing or IFN-γ release in TIL-retaining, microtumor, and micro-organosphere systems [18,55,83] (proof-of-concept). ② Integrate multiple determinants into one functional response. A microsatellite-stable but POLE-mutated, high-TMB colorectal tumor responds, and a PD-L1-negative tumor shows functional reactivity, results that no single biomarker predicts [18,83] (proof-of-concept). ③ Reproduce resistance: preservation-specific local Treg expansion after PD-1 blockade, and REG4-associated resistance reversed on knockout [19,85] (proof-of-concept, mechanistic). ④ Approximate clinical response rates: head and neck air–liquid interface (ALI) organoids expanding tumor-reactive CD8^+^ T cells [28], set against a real-world benchmark of near-complete response in mismatch-repair-deficient colon cancer [91]. Breast cancer remains a blank, supported only by engineering groundwork [86,87] rather than direct prediction (emerging). (Center) Static classifiers (PD-L1 immunohistochemistry, TMB, and microsatellite status) are fast and standardized but give a molecular snapshot; organoid functional readouts are dynamic and integrative but slower, less standardized, and culture-dependent. The two converge to refine stratification, with greatest value for the microsatellite-stable, PD-L1-low, intermediate-TMB non-responder majority; no prospective head-to-head comparison is yet reported. (Right) The validation loop: construction strategy → predictive readout → prospective paired validation → identification of which construction captures checkpoint sensitivity → back to construction, moving the field from proof-of-concept avatars toward validated instruments that complement rather than replace molecular biomarkers. ALI, air–liquid interface; ICB, immune checkpoint blockade; IFN-γ, interferon-γ; MSI, microsatellite instability; MSS, microsatellite stable; PD-1, programmed cell death protein 1; PD-L1, programmed death-ligand 1; TIL, tumor-infiltrating lymphocyte; TMB, tumor mutational burden; Treg, regulatory T cell.

Put together, immune-competent organoids clear the preconditions for checkpoint prediction but have not reached the bar of validated predictors. Every current match to outcome is retrospective or cohort-descriptive, and prospective studies treating patients by an organoid readout are all but absent. The coverage is also uneven: tumors yielding large resection specimens with an accessible infiltrate are heavily represented, whereas malignancies sampled mainly by a small biopsy are sparsely covered. The construction methods are, in principle, transferable, since neither the ALI culture nor co-culture is histology-specific. What does not transfer automatically is the tissue input requirement, the niche factor formulation that each tumor type needs, and the checkpoint response benchmark against which a readout would be judged (Figure 3).

## 8. Organoid Functional Readouts Versus Established Biomarkers

To place organoid functional prediction within clinical practice, the comparison to make is with the molecular biomarkers now guiding checkpoint therapy. The established biomarkers are static measurements of a tumor’s molecular state; an organoid readout is a functional measurement of how tumor and immune cells behave together. The question is whether functional organoid readouts can fill the deficiencies just described, and what their strengths and weaknesses are for clinical application.

An organoid functional readout departs from every static measure by folding their separate dimensions into one behavioral output. Because a checkpoint effect depends at once on antigen presentation, T-cell recognition, and the suppressive pull of stroma and myeloid cells, the readout reflects their combined result rather than any single axis. The microsatellite-stable, POLE-mutated colorectal case is one instance [83], and functional readouts can also register a response in PD-L1-negative tumors [18]. Molecular biomarkers are fast, standardized, and measured straight off clinical tissue; organoid assays are slower, less standardized, and hostage to a successful culture, but they report the functional outcome that no molecular snapshot holds.

For the near term, the fitting role is a complementary layer that sharpens the biomarker-based stratification. Fast, tissue-based, standardized molecular markers suit first-line stratification and trial enrollment. The tumor mutational burden defined the biomarker-selected cohort in the tissue-agnostic KEYNOTE-158 analysis in just this way [100]. A functional organoid readout would then serve as a second-line test for the cases that those markers classify poorly, namely, the microsatellite-stable, PD-L1-low, intermediate-burden group identified in Section 1. No study has prospectively compared the organoid functional readouts head-to-head against the PD-L1, tumor mutational burden, or microsatellite status in the same patients, with the response as the endpoint. This sequential framing therefore stays a well-motivated hypothesis rather than a demonstrated advance. Claims that functional assays outperform molecular biomarkers are not supported by the current evidence.

### Complement to Animal Models Rather than Replacement

A second positioning question bears as directly on how these platforms should be used: do immune-competent organoids replace animal and xenograft models, or complement them? The asymmetry is specific. The checkpoint response depends on a tumor meeting the immune system of the patient carrying it, and an immune-competent organoid supplies that directly. Obtaining it in vivo instead requires host humanization, or a mouse-derived immune system that is not the patient’s own [10]. What animal models retain is much of what the preceding sections listed as absent from a dish. That list includes a perfused vasculature and endothelial barrier, immune cell trafficking and lymph node priming, systemic pharmacokinetics, and metastatic sites. They also offer an experimental window of weeks to months, rather than the days to few weeks that a preserved immune compartment stays functional.

On the present evidence, the defensible position is complementary and sequential rather than substitutive. Organoid readouts suit questions that are patient-specific, mechanistic, and answerable within a short window, and they help narrow the conditions that warrant in vivo testing. Questions turning on a circulating immune system, drug distribution, or a survival endpoint remain in vivo questions. The on-chip anti-PD-1 readout that corresponded to subsequent in vivo efficacy was informative because both were performed, one prioritizing what the other tested [53]. Regulatory frameworks for non-animal methods are moving in a direction that would admit qualified in vitro systems for defined purposes (Section 9). That does not establish equivalence for response prediction, and no head-to-head comparison against an in vivo predictor has been reported. Immune-competent organoids are best placed as a front-of-line, patient-specific functional layer that complements rather than replaces in vivo models, and both positioning claims rest on one set of unmet requirements: a reproducible derivation, comparable readouts, and a prospective paired validation.

## 9. Opportunities and Challenges

Between immune-competent organoids and clinical use lies a connected chain: is the model reproducible, is the evidence sufficient, does a regulatory path exist, and is the platform accessible? Each link constrains the next. Table 4 sets out, link by link, what these organoids can already do, the evidence level reached, what remains unsolved, and the measurement that would settle each open question. Section 9.1 takes the capabilities, Section 9.2 the obstacles.

### 9.1. Opportunities and Applications

Four uses account for most of what immune-competent organoids currently offer, and separating them shows which rests on what grade of evidence. The first of these is biomarker discovery. These models expose tumor and immune cells to a defined perturbation and report the result, so they identify correlates that a tissue section cannot. Paired single-cell and repertoire sequencing shows which clones expand under the checkpoint blockade [15]. Fragment perturbation has also pointed to tumor-resident T cells and tertiary lymphoid structures as correlates of the reactivation capacity [68]. The second is combination screening. A model containing both compartments allows an immunomodulatory agent to be tested with a cytotoxic or targeted one in the same well. That design is how chemotherapy-induced immunogenic signals were shown to repolarize macrophages in a reconstructed colorectal niche [46], and droplet and device formats extend the logic to scale [53,55].

The third is resistance mechanism discovery, which is strongest in mechanistic rather than predictive terms. Ex vivo checkpoint exposure has reproduced the adaptive resistance arising within the model through local regulatory T-cell expansion [19], and it has implicated a molecular determinant whose removal restored sensitivity [85]. These results explain a resistance route without establishing that an ex vivo signal anticipates clinical progression. The fourth, furthest from routine use, is individualized treatment selection: functional readouts have resolved cases that static classifiers assign incorrectly [18,83], the basis for the second-line complementary role set out in Section 8. That role remains an emerging proposal, since every current match to a clinical outcome is retrospective and no patient has been treated according to an organoid readout.

### 9.2. Current Challenges

No standardized protocol governs how immune-competent organoids are derived, cultured, or assayed, and co-culture systems especially lack agreed procedures for effector preparation, geometry, and functional endpoints [101,102]. The trouble starts at derivation, where the establishment rates swing widely between centers, which is why expert-consensus efforts have named standardized workflows an unmet need [103] and why tumor-specific standards now set explicit quantitative criteria for successful establishment [104]. The endogenous immune compartment lasts only days to a few weeks, and the throughput sits far below that of epithelial organoid biobanks. Absent shared standards, the functional results cannot be pooled toward the thresholds that prediction demands.

Even where a model is technically sound, the supporting evidence is thin. Studies linking organoid readouts to the checkpoint response are almost, without exception, small, retrospective, and cross-sectional, and reviews of the field are careful to say the question is still formally unresolved [24,107]. Chemotherapy offers the closest precedent: a prospective trial found colorectal organoids predicted the response to one regimen accurately but failed for another, so the predictive validity must be earned context by context [105]. Immunotherapy prediction is biologically more tangled and has had no comparable prospective test.

Closing that gap means bringing the model into formal clinical translation pathways, which are only taking shape. Co-clinical designs test a patient’s organoid alongside their treatment, and they are one way to accumulate the paired data that prospective validation needs. Such designs have matched a prospective colorectal trial to patient-derived organoids to find the resistance biomarkers for targeted therapy [106]. To our knowledge, none has yet been applied to organoid-based immunotherapy prediction. The fit is good; since the readout and clinical response come from the same patient, every case yields one matched pair that can be scored blind, and a low throughput is tolerable because patients enroll one at a time. At scale, this would make the organoid a decision-support tool rather than a research assay, which would call for qualification against regulatory expectations [108]. The wider climate is moving that way, with frameworks for qualifying microphysiological systems under development [109] and legislation letting non-animal methods support drug applications [110]. These routes are forming, not settled, and none yet qualifies an immune-competent organoid as a validated predictor.

Immune-competent models require fresh, adequately sized surgical specimens with an intact immune infiltrate, a requirement that limits sampling and disadvantages small biopsies and hard-to-reach tumors. Biobanks built from such specimens need harmonized processing and data standards before a multi-center work becomes possible, and their governance raises unsettled consent and equity questions. These access limits feed back on the earlier ones, since a platform confined to a few centers cannot generate the large, harmonized, prospectively validated datasets that standardization and evidence-building require.

## 10. Conclusions and Future Perspectives

The central lesson of immune-competent tumor organoids is that construction determines prediction. Whether a model keeps an endogenous infiltrate or rebuilds the immunity from defined effectors sets which parts of the checkpoint biology it can report on, which makes organoid immunoprofiling a family of platforms. What it offers is a functional, patient-specific readout that folds the antigen presentation, T-cell recognition, and stromal-myeloid suppression into one output. That output resolves individual responses that static biomarkers misclassify, reproduces the adaptive resistance, and may extend the prediction to the microsatellite-stable tumors the current markers pass over.

Analytical methods are developing alongside, but at a remove from, the models reviewed here, as the subsection on multi-omic and computational layers in Section 6 sets out: extending organoid-trained algorithms to checkpoint readouts remains a prospect rather than a result [78,79,80].

Progress must come at three linked levels: standardized derivation and a longer immune viability to make results reproducible, shared potency metrics and spatial integration to make readouts poolable, and a prospective paired validation to test whether either translates. Only through such a closed loop can immune-competent organoids move toward validated instruments for individualizing immunotherapy [111], complementing rather than replacing the biomarkers that guide treatment today.

Four milestones would close that loop. All are phrased as quantities a study could measure and report, and Table 4 pairs each with the open question it answers:

- Every immune-competent platform should report the same minimum set: establishment success against a defined denominator, plus immune cell viability and infiltrate retention at the stated time points. The immune composition, clonal structure, and activation state must also be compared between fresh tissue and the cultured model, and reported as a change over time rather than a single endpoint [15,16,17].
- Potency, then, needs a metric everyone shares. Cell-therapy manufacturing supplies the template: killing normalized to a defined effector-to-target ratio, activity referenced to a common benchmark instead of laboratory-specific units, every endpoint tied to a stated mechanism of action [71,72].
- Where regulatory T cells and cancer-associated fibroblasts sit relative to effector cells should be measured inside the cultured model itself, not inferred from bulk suppression. Only then does the geometry of a reconstructed microenvironment stop being an assumption and become an observation [66,75].
- Last comes the prospective paired validation, with the threshold fixed in advance. A co-clinical design would give each enrolled patient one matched readout-and-outcome pair, scored blind. Baseline resistance readouts would be paired with progression-free survival. The organoid readout would then be compared, in that same cohort, against the PD-L1 immunohistochemistry, tumor mutational burden, and microsatellite status, with the clinical response as the endpoint [106].

None of the four asks for a technique that does not exist; each asks for a quantity to be defined, reported, and paired with an outcome. What makes that path tractable is the construction-to-prediction principle itself. It tells the field to match each construction choice to the predictive question it is meant to answer. It also asks that the construction be reported in enough detail for others to test the same claim.

## Figures and Tables

**Table 3:** Evidence for organoid-based prediction of immune checkpoint blockade response, organized by predictive capability and graded by evidence level.

Predictive Capability	Tumor Type(s)	Model/Construction	Checkpoint Agent or Effector	Functional Readout	Key Finding	Evidence Level	[Ref.]
① Register a checkpoint effect	Sarcoma, melanoma, others	PDTO	Anti-PD-1/anti-PD-L1	Organoid killing/viability	Feasibility of modeling an ICB response in patient-derived organoids	Feasibility	[82]
① Register a checkpoint effect	PD-L1-negative and other tumors	TIL-retaining PDTO	Nivolumab	Dose-dependent killing; IFN-γ	Dose-dependent tumor shrinkage; PD-L1-negative tumors also respond	Proof-of-concept	[18]
① Register a checkpoint effect	Colorectal	Microtumor	Pembrolizumab	IFN-γ secretion	Functional IFN-γ response to checkpoint blockade	Proof-of-concept	[83]
① Register a checkpoint effect	Multiple	Micro-organosphere	ICB/drug panel	Rapid drug sensitivity	Rapid, scalable functional sensitivity testing	Proof-of-concept	[55]
① Register a checkpoint effect	Multiple	Organoid spheroid; mini-tumor chip	Anti-PD-1	Killing; on-chip response	Reproduces PD-1-sensitive vs. -resistant behavior; on-chip response tracks in vivo efficacy	Proof-of-concept	[52,53]
① Register a checkpoint effect	Gastric	PDTO	HER2/PD-L1 axis	Immune evasion readout	Models HER2–PD-L1-associated immune evasion	Proof-of-concept	[84]
② Integrate multiple determinants into one functional response	Colorectal	Microtumor	Pembrolizumab	IFN-γ/killing	MSS but POLE-mutant/high-TMB case still responds; no single biomarker predicts it	Proof-of-concept (case-level integration)	[83]
② Integrate multiple determinants into one functional response	PD-L1-negative tumors	TIL-retaining PDTO	Nivolumab	Dose-dependent killing	Functional response despite negative static biomarker	Proof-of-concept (case-level integration)	[18]
③ Reproduce resistance	Colorectal	ALI preservation	PD-1 blockade	Local Treg expansion	Post-treatment local Treg expansion (preservation-specific; not reproduced in co-culture)	Proof-of-concept (mechanistic)	[19]
③ Reproduce resistance	Colorectal	PDTO	ICB context	Response/re-sensitization	REG4-associated resistance; knockout restores sensitivity	Proof-of-concept (mechanistic)	[85]
④ Approximate clinical response rates	Head and neck	ALI expansion	Pembrolizumab	Reactive CD8^+^ expansion	Expands tumor-reactive CD8^+^ T cells; aligning to clinical ORR is a near-term objective	Emerging	[28]
④ Approximate clinical response rates	Breast	On-chip/scaffold engineering; endogenous immune baseline	—	Engineering groundwork; immune baseline	No peer-reviewed direct ICB prediction; only engineering groundwork and endogenous immune baseline established	Blank/proving ground	[86,87,88,89,90]

Evidence levels follow the four-level scale defined in Section 2. The circled numerals ①–④ label the four predictive capabilities introduced in Section 7. Evidence for ICB response prediction is uniformly emerging or proof-of-concept; cohort correlations available only as conference abstracts are graded as low-level evidence pending peer review; breast cancer is reported as a blank/proving ground. This table lists organoid-based evidence only. Real-world clinical benchmarks are discussed in the text as external reference targets against which organoid readouts must eventually be calibrated, and they are deliberately not listed as organoid evidence. One such benchmark is the near-complete pathological response to neoadjuvant PD-1 blockade in mismatch-repair-deficient colon cancer [91]. A cohort-level correlation in gastric cancer available only as a conference abstract is likewise noted in the text rather than tabulated. Abbreviations: ALI, air–liquid interface; ICB, immune checkpoint blockade; IFN-γ, interferon-γ; MSS, microsatellite stable; ORR, objective response rate; PD-1, programmed cell death protein 1; PD-L1, programmed death-ligand 1; PDTO, patient-derived tumor organoid; TIL, tumor-infiltrating lymphocyte; TMB, tumor mutational burden; Treg, regulatory T cell.

**Table 4:** What immune-competent tumor organoids can already do and what remains unsolved, with the evidence level reached and the readout that would settle each open question.

Capability	What Is Already Achievable	Evidence Level	What Remains Unsolved	Readout That Would Settle It
Immune-competent derivation	Fragment-based culture retains T, B, NK, and myeloid cells together with the parental TCR repertoire, and co-culture regenerates tumor-reactive CD8^+^ T cells from blood in roughly a third to a half of samples [15,16,17]	Proof-of-concept	Whether the retained cells keep their original frequency, clonal diversity, and functional state, rather than merely remaining detectable	Immune composition, clonal structure and activation state compared between fresh tissue and the cultured model at defined time points, reported as a change rather than an endpoint
Registering a checkpoint effect	Dose-dependent, checkpoint-dependent killing and IFN-γ release, including in PD-L1-negative tumors, on a timescale compatible with clinical decisions [18,55,83]	Proof-of-concept	Whether the magnitude of an ex vivo effect maps onto a clinical response category, rather than merely differing from control	Ex vivo effect size paired with the same patient’s response and scored against a threshold fixed in advance
Integrating several determinants	One functional output that reflects antigen presentation, T-cell recognition, and stromal-myeloid suppression together, resolving individual cases that static classifiers misassign [18,83]	Proof-of-concept	Whether that integration adds predictive information beyond the established classifiers when both are measured in the same patients; this has not been tested in either direction	Prospective comparison against PD-L1 immunohistochemistry, tumor mutational burden, and microsatellite status in one cohort, with clinical response as the endpoint
Reproducing resistance	Preservation-specific local Treg expansion after PD-1 blockade, and REG4-associated resistance that was reversed on knockout [19,85]	Proof-of-concept, mechanistic	Whether an ex vivo resistance readout anticipates clinical progression rather than describing it retrospectively	Baseline resistance readout paired with progression-free survival in the same patients
Duration of immune competence	Endogenous immune cells detectable and functional for roughly 10 to 30 days, extended by protocol optimization and by continuous perfusion [15,31,52,53,54]	Feasibility	No window long enough to follow a treatment course; no systemic compartment, no pharmacokinetics, and no representation of newly emerging lesions	Immune viability, infiltrate retention, and functional state reported at fixed time points as a minimum data set for every platform
Standardized potency	Cell-therapy manufacturing supplies the template: validated flow-cytometric killing assays covering specificity, linearity, accuracy, and precision [71,72]	Blank for immune-competent organoids	Organoid readouts use laboratory-specific units, uncalibrated effector-to-target ratios and heterogeneous endpoints, so results cannot be pooled toward a common threshold [69,73,74]	Killing normalized to a defined effector-to-target ratio, activity referenced to a shared benchmark rather than arbitrary units, and endpoints tied to a stated mechanism of action
Spatial resolution	Multiplexed imaging of intact tumors shows that coordinated cellular neighborhoods, not densities alone, govern antitumoral immunity, and pairing spatial methods with three-dimensional models has been proposed [66,75]	Blank for immune-competent organoids	Spatial omics have not been applied inside an immune-competent organoid, so the geometry of reconstructed suppression is inferred rather than measured	Position of Treg and CAF populations relative to effector cells, measured within the cultured model itself
Reproducibility and throughput	Fragment size standardized by microdissection, up to 960 mini-tumors generated per chip, and establishment above 97% from 10 mg of tissue in cluster format [32,51,53]	Feasibility	No agreed procedure for effector preparation, co-culture geometry, or functional endpoints, and establishment rates that vary widely between centers [101,102,103,104]	A minimal reporting set: establishment success against a defined denominator, immune cell viability, and infiltrate retention
Clinical validation	Qualitative concordance in small retrospective series, and one on-chip readout related to a subsequent in vivo outcome [53]	Emerging	No prospective study has treated patients according to an organoid readout, and the chemotherapy precedent shows that predictive validity must be earned regimen by regimen [105]	A co-clinical design yielding one matched readout and outcome pair per enrolled patient, with the assay scored blind to outcome [106]
Tumor-type coverage	Most functional ICB-prediction data come from colorectal cancer, with corroborating observations in head and neck, gastric, renal, and lung cohorts [28,91]	Emerging, and blank for breast cancer	Breast cancer has substantial engineering groundwork but no peer-reviewed predictive demonstration, and tumors sampled mainly by small biopsy remain sparsely covered [86,87,88,89,90,98,99]	Per-indication establishment rate from biopsy-scale tissue, together with a stated checkpoint response benchmark for that indication

Evidence levels are the four-level scale defined in Section 2 and describe how directly the cited work bears on predicting checkpoint response, not the quality of the studies. “Blank” is used where the clinical or methodological need is established but no peer-reviewed demonstration exists in an immune-competent organoid. The final column states the measurement that would move each row from the unsolved to the achievable column; these are the milestones returned to in Section 10. Abbreviations: CAF, cancer-associated fibroblast; ICB, immune checkpoint blockade; IFN-γ, interferon-γ; NK, natural killer; PD-1, programmed cell death protein 1; PD-L1, programmed death-ligand 1; TCR, T-cell receptor; Treg, regulatory T cell.

## Data Availability

No new data were created or analyzed in this study. Data sharing is not applicable to this article.

## Abbreviations

The following abbreviations are used in this manuscript:

ALI	air–liquid interface
CAF	cancer-associated fibroblast
CAR	chimeric antigen receptor
dMMR	mismatch-repair deficient
ICB	immune checkpoint blockade
IFN-γ	interferon-γ
iPSC	induced pluripotent stem cell
MSS	microsatellite stable
NK	natural killer
ORR	objective response rate
PBL	peripheral blood lymphocyte
PDTO	patient-derived tumor organoid
TCR	T-cell receptor
TGF-β	transforming growth factor-β
TIL	tumor-infiltrating lymphocyte
TMB	tumor mutational burden
TNBC	triple-negative breast cancer
TNF-α	tumor necrosis factor-α
Treg	regulatory T cell
VEGF	vascular endothelial growth factor
